# The monosaccharide transporter gene family in land plants is ancient and shows differential subfamily expression and expansion across lineages

**DOI:** 10.1186/1471-2148-6-64

**Published:** 2006-08-21

**Authors:** Deborah A Johnson, Jeffrey P Hill, Michael A Thomas

**Affiliations:** 1 Department of Biological Sciences, Idaho State University, Campus Box 8007, Pocatello, ID, USA

## Abstract

**Background:**

In plants, tandem, segmental and whole-genome duplications are prevalent, resulting in large numbers of duplicate loci. Recent studies suggest that duplicate genes diverge predominantly through the partitioning of expression and that breadth of gene expression is related to the rate of gene duplication and protein sequence evolution.

Here, we utilize expressed sequence tag (EST) data to study gene duplication and expression patterns in the monosaccharide transporter (MST) gene family across the land plants. In *Arabidopsis*, there are 53 MST genes that form seven distinct subfamilies. We created profile hidden Markov models of each subfamily and searched EST databases representing diverse land plant lineages to address the following questions: 1) Are homologs of each *Arabidopsis *subfamily present in the earliest land plants? 2) Do expression patterns among subfamilies and individual genes within subfamilies differ across lineages? 3) Has gene duplication within each lineage resulted in lineage-specific expansion patterns? We also looked for correlations between relative EST database representation in *Arabidopsis *and similarity to orthologs in early lineages.

**Results:**

Homologs of all seven MST subfamilies were present in land plants at least 400 million years ago. Subfamily expression levels vary across lineages with greater relative expression of the STP, ERD6-like, INT and PLT subfamilies in the vascular plants. In the large EST databases of the moss, gymnosperm, monocot and eudicot lineages, EST contig construction reveals that MST subfamilies have experienced lineage-specific expansions. Large subfamily expansions appear to be due to multiple gene duplications arising from single ancestral genes. In *Arabidopsis*, one or a few genes within most subfamilies have much higher EST database representation than others. Most highly represented (broadly expressed) genes in *Arabidopsis *have best match orthologs in early divergent lineages.

**Conclusion:**

The seven subfamilies of the *Arabidopsis *MST gene family are ancient in land plants and show differential subfamily expression and lineage-specific subfamily expansions. Patterns of gene expression in *Arabidopsis *and correlation of highly represented genes with best match homologs in early lineages suggests that broadly expressed genes are often highly conserved, and that most genes have more limited expression.

## Background

Large proportions of genes within genomes are members of hierarchical gene families and superfamilies. Gene families appear to evolve through a combination of tandem, segmental and whole genome duplication (polyploidy) events. A number of researchers in the first half of the twentieth century observed relationships between chromosome duplications and morphological variation [1]. In 1970, Ohno [2] argued that, because natural selection is inherently conservative, major genetic novelty can arise only through gene duplication events where purifying selection is relaxed on one of the duplicates. The classical model of the fates of duplicate genes [2-4] predicts that most gene duplicates are lost due to deleterious mutations and that new function arises only with rare beneficial mutations resulting from neutral processes.

More recent theoretical and empirical work suggests that gene duplicates are retained more frequently than the classical model permits and that new function or expression arises through the processes of neo- and subfunctionalization [5,6]. In subfunctionalization, expression or function present in a progenitor gene is partitioned between daughter genes through complementary mutations to regulatory or coding regions [7]. In neofunctionalization, related or novel function may arise in one of the duplicates through initial relaxation of purifying selection with accumulation of mutations conferring new function under either neutral or positive selection. Partitioning of expression appears to be the most common fate of a fixed gene duplicate [8,9] and it appears to happen relatively rapidly after duplication [9]. However, many gene duplicate pairs appear to evolve slowly, suggesting that buffering of crucial functions may be important after gene duplication events [10].

Plant genomes contain large fractions of duplicate loci due to the frequent occurrence of segmental duplications and polyploidy events. Following a polyploidy event, there is a rapid loss of duplicate loci in the transition to functional diploidy and the remaining duplicate loci undergo rapid functional divergence [11]. Recent genome-scale studies indicate that some types of duplicate genes are retained at higher frequencies than others [12-14], that highly conserved genes are duplicated and retained more frequently than more rapidly evolving genes [15], and that rates of protein evolution may be related to expression levels [16,17] and patterns, with genes expressed in multiple tissues under stronger purifying selection [18,19].

In this study, we investigate the monosaccharide transporter (MST) gene family in land plants. MSTs are found in all three domains of life, have fundamental importance in carbohydrate flux and are highly conserved across lineages. All MST proteins are characterized by 12 hydrophobic membrane-spanning domains separated by interconnecting cytoplasmic and extracellular loops, with cytoplasmic N- and C-terminal domains [20]. This highly conserved protein structure provides a strong signature for identification of putative MSTs in translated DNA sequence data, such as ESTs. Most plant MST genes characterized to date show expression in sink tissues and are thought to function in the uptake of simple sugars from the apoplast after phloem-unloading and hydrolysis of sucrose by co-expressed cell wall invertases [21,22]. Most have been shown to be H^+^-sugar symporters localized in the plasma membrane (see references below).

**Figure 1:**
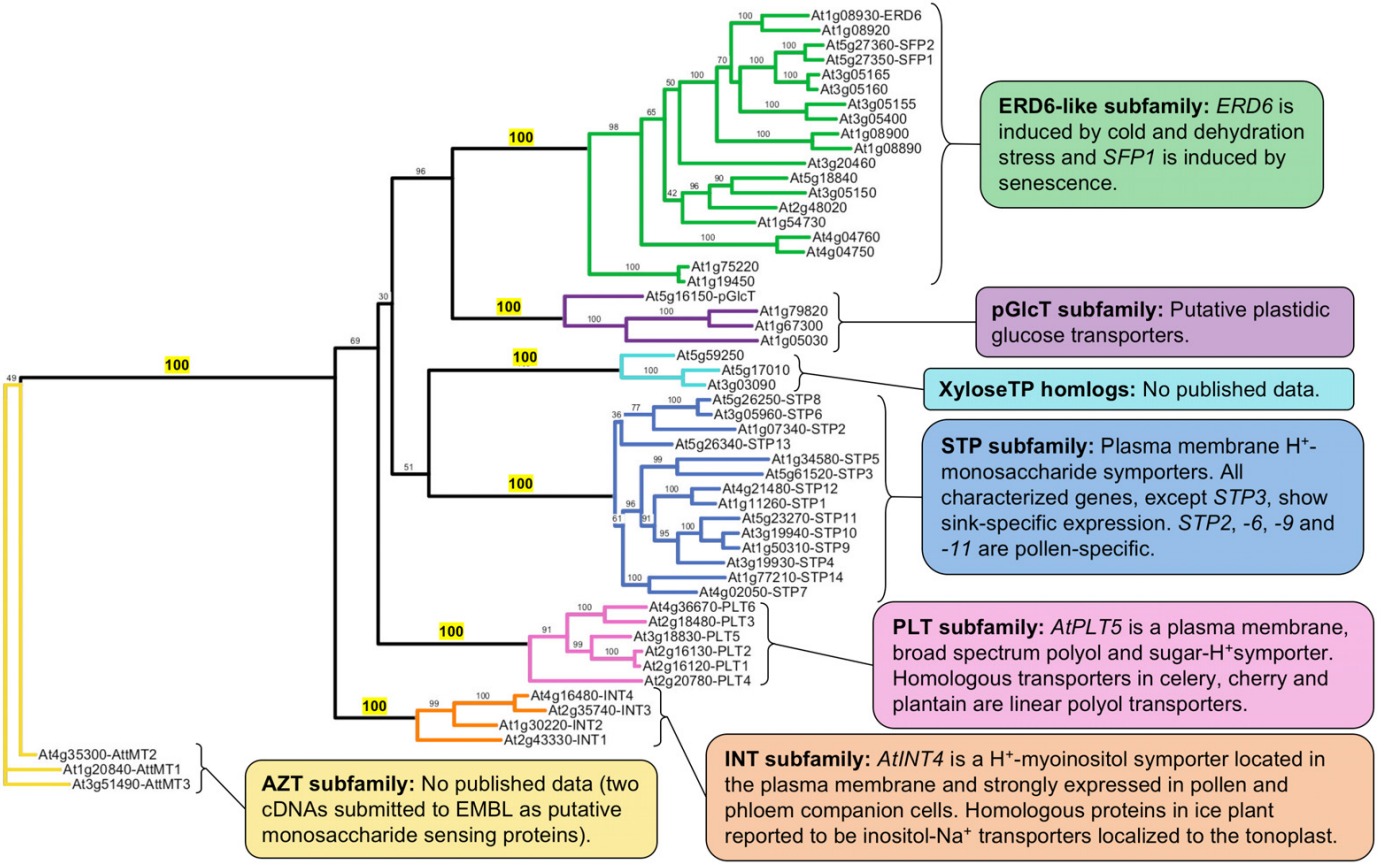
**Maximum likelihood phylogeny of Arabidopsis MSTproteins**. An unrooted phylogeny of the 53 *Arabidopsis *MST protein sequences inferred using maximum likelihood. The tree was produced using PHYML with the JTT amino acid substitution model, a discrete gamma model with four categories and an estimated shape parameter of 1.385. Bootstrapping was performed with 100 replicates. Bootstrap values for each subfamily clade are highlighted in yellow. Call-outs show available information about the function and expression of some MST genes, from *Arabidopsis *and other taxa, within each subfamily.

Previous analysis of the *Arabidopsis thaliana *genome reveals 53 MST genes that cluster into seven subfamilies on phylogenetic analysis (Figure 1) [23]. To date, less than a dozen of these 53 genes have been characterized as to function and/or expression. The STP subfamily is the best studied, with published reports on seven of the 14 genes [24-30]. Only a handful of genes in the other subfamilies, *AtERD6 *[31], *AtSFP1 *[32], *AtpGlcT *[33], *AtPLT5 *[34], and *AtINT4 *[35] have been studied. However, a number of MSTs in green algae [36] and other higher plants [20,33,37-40] have been investigated, contributing to our understanding of the functional diversity of this gene family in the green plants as a whole. Because some of these proteins have been documented to transport sugar alcohols, this gene family is named the MST(-like) gene family on the TAIR website [41]. For simplicity, we will refer to all genes in the MST(-like) family as MST genes.

The great diversity of land plants on Earth today is strongly supported as a monophyletic clade [42,43]. As such, it presents an opportunity to study gene family evolution in major lineages that differ greatly in structural complexity and life histories. Land plants consist broadly of primitive nonvascular and more complex vascular plants. The small, structurally simple nonvascular plants are composed of three major groups, the liverworts, mosses, and hornworts, collectively known as "the bryophytes." In bryophytes, the small diploid sporophyte is epiphytic on the dominant, leafy haploid gametophyte. Lycophytes are the earliest divergent vascular plants represented today by only a few extant lineages, the club mosses (Lycopodiaceae), spike mosses (Selaginellaceae), and quillworts (Isoetaceae). The pteridophytes are composed of three lineages of ferns (Ophioglossaceae, Marattiales, and Polypodiales), the horsetails (*Equisetum*) and whisk ferns (Psilotaceae) that form a monophyletic group which is sister to the seed plants [44]. Seed plants are composed of four groups of gymnosperms (conifers, cycads, ginkgos and gnetophytes) and the angiosperms (flowering plants). All land plants exhibit alternation of generations, the formation of a multicellular body in both the haploid and diploid phase of the life cycle. However, in contrast to the non-vascular bryophytes, all vascular plant lineages are characterized by life histories in which the diploid sporophyte is dominant and the haploid gametophyte is much reduced in size. In the seed plants (especially the flowering plants), the gametophytes are most reduced in size and nutritionally dependent upon the sporophyte.

Because the common ancestor of land plants is inferred to have had a haploid-dominant life cycle [42,45,46], hypotheses regarding gene expression in the simple gametophyte versus the complex sporophyte have focused around the idea that sporophyte genes were 'recruited' from the haploid gametophyte genome in the early evolution of land plants, resulting in overlapping expression of identical genes in both generations [47-49]. Hypotheses that integrate the potentially significant role of gene duplication and divergence in the early evolution of the complex sporophyte have begun to be developed and tested [45,50]. The study of specific gene families in the earliest land plant lineages with dominant or independent gametophytes may help to answer these questions.

Analyzing a complete genome sequence is the only method whereby all members of a gene family can be identified with certainty. At the time of this study, complete plant genome sequences are available for *Arabidopsis thaliana *and *Oryza sativa *(rice). However, large EST databases (>100,000 ESTs) derived from multiple tissues, developmental stages and experimental conditions represent a resource for studying the genomes of species with unsequenced genomes through their transcriptomes. Large EST databases are available for many important crop plants across the monocot and eudicot flowering plants. However, only two large EST databases are available for earlier divergent land plants: the moss *Physcomitrella patens *[49] and the gymnosperm *Pinus taeda *(loblolly pine) [51]. Small EST databases exist for several species belonging to other major early land plant lineages including a liverwort, *Marchantia polymorpha*, a lycophyte, *Selaginella lepidophylla*, and the fern *Ceratopteris richardii*. Each of these small databases is derived from limited tissue types, the *Marchantia *library constructed from sex organ tissues (gametophyte), the *Selaginella *database from dessicated frond (sporophyte), and the *Ceratopteris *library from germinating spores (gametophyte). We searched the large EST databases of *Zea mays *(corn), *Lycopersicon esculentum *(tomato), *Pinus taeda*, *Physcomitrella patens *and *Arabidopsis thaliana*. We also searched the small *Marchantia*, *Selaginella *and *Ceratopteris *EST databases described above.

To search these databases, we constructed profile hidden Markov models (HMMs) of each MST subfamily. Profile HMMs are probabilistic models representing an alignment of multiple amino acid sequences that are very effective at identifying related sequences [52]. To build a profile HMM of a particular protein family, a multiple sequence alignment containing sequences from as diverse an assembly of species as possible is created in order to properly represent both the conserved and divergent regions across the protein family. The resulting profile HMMs (or consensus protein sequences created from them) can be used to search EST databases with software such as the Wise2 package [53].

Given that MSTs are ubiquitous across all three domains of life and that homologs of each of the seven *Arabidopsis *subfamilies have been found in many vascular plant species as well as the green alga *Chlorella kessleri*, we hypothesize that the seven subfamilies of MST genes identified in *Arabidopsis *are ancient, with ancestral homologs of each subfamily likely present in the earliest land plants. Second, given the prevalence of individual gene, segmental chromosome and whole genome duplications within the land plants, the MST subfamilies are likely to have unique expansion patterns within lineages. Third, if partitioning of expression is the most prevalent fate of duplicate genes, then we would expect to find unique patterns of MST gene expression across lineages. Last, if broadly expressed genes are more conserved than narrowly expressed genes, then *Arabidopsis *genes with broad expression should be most similar to orthologs in the earliest lineages. We infer breadth of expression of *Arabidopsis *MST genes based on their relative representation, within subfamily, in the combined EST database along with an evaluation of microarray data.

To investigate these questions, our study consisted of the following analyses: (1) construction of a statistically robust phylogenetic tree of *Arabidopsis *MST proteins; (2) mapping of each *Arabidopsis *MST gene to determine segmental and tandem gene duplications; (3) construction of profile HMMs and consensus protein sequences for each MST subfamily; (4) a search of the large *Arabidopsis *EST database to determine relative representation of each MST gene (and to provide a comparison for evaluating the effectiveness of our profile HMMs at identifying ESTs in other species); (5) a search of EST databases of other major land plant lineages (described above) for ESTs belonging to each MST subfamily; (6) creation of EST contigs to infer the number of expressed MST loci present in each large EST database; and (7) correlation of *Arabidopsis *MST genes with high EST database representation with best match homologs in early divergent lineages.

## Results

### Phylogenetic analysis and mapping of the MST gene family in *Arabidopsis*

Phylogenetic analysis of the 53 *Arabidopsis *MST protein sequences using the maximum likelihood (ML) method (Figure 1) revealed a phylogeny in agreement with the phylogeny posted on the Arabidopsis Sugar Transporter homepage [23], with one notable exception: In our ML tree, the *AtSTP13 *protein clusters at the base of the subclade containing the *AtSTP2*, *-6 *and *-8 *genes, rather than grouping with the *AtSTP14 *and *AtSTP7 *genes. The bootstrap value for this arrangement is quite low at 36, indicating an unresolved node. However, mapping the STP genes on the *Arabidopsis *chromosomes (Additional file 1) supports a close relationship between *AtSTP6 *and *AtSTP13 *as a result of a segmental duplication event involving these two genes. Across the tree, most bootstrap values were 90 or higher, with all seven nodes at the base of each subfamily clade having bootstrap values of 100. A consensus maximum parsimony (MP) tree of all 53 MST protein sequences with 10,000 bootstrap replicates revealed a topology that was essentially the same as the ML topology with similar support values among most genes (not shown). Nodes with low bootstrap support values on the ML tree are represented by polytomies on the MP tree. In our MP tree, the *AtSTP13 *gene forms a polytomy with four other STP gene groups, including the *AtSTP7*-*14 *and *AtSTP2*-*6*-*8 *groups. The chromosome map of all 53 MST genes (Additional file 1) reveals six regions of tandem gene duplications, four of which involve ERD6-like genes. Segmental duplications are present in all subfamilies except the pGlcT subfamily.

### Construction of profile hidden Markov models and consensus sequences

We searched the Protein Families (pfam) database [54] for all full-length or nearly full-length non-*Arabidopsis *MST genes within the viridiplantae clade. This resulted in a set of 62 MST genes from 25 different species (Additional file 2). Of the 62 MST genes, 30 were from monocot species, 31 from eudicot species, and one from the gymnosperm *Picea abies*. These were combined with the 53 *Arabidopsis *MST genes [23], three *Chlorella kessleri *hexose transporters (*CkHUP1-3*), and two partial *Ceratopteris richardii *MST genes (*CrMST1-2)*, for a total of 120 MST genes. Each of the MST subfamily profile HMMs is available as a separate file (Additional files 3, 4, 5, 6, 7, 8, 9). An alignment of the consensus sequences generated from each subfamily profile HMM shows the AZT subfamily to have a large central loop, the INT subfamily to have an expanded region from amino acids 749–824, and the XyloseTP homologs to have a long N-terminal domain (Additional file 10). Three-dimensional protein structures are not available for any of these proteins.

### Summary of EST database search results

The percentage of identified MST genes in each EST database varies from 0.05% in *Lycopersicon *to 0.28% in *Arabidopsis*, with an average of 0.09% (Table 1). In the large EST databases, differences in relative proportions of ESTs from each of the seven subfamilies also vary across the land plant lineages. In *Physcomitrella*, the AZT and pGlcT subfamilies each represent 37.5% of the total MST ESTs, for a total of 75%. However, in the vascular and flowering plants, especially in the dicot lineages, the STP and ERD6-like subfamilies appear to have increased expression levels relative to the other subfamilies, with the STP ESTs comprising 43.9% of total MST ESTs in *Arabidopsis *and the ERD6-like ESTs comprising 35.5% of total MST ESTs in *Lycopersicon*. In the very small EST databases of the early land plant lineages, percentages of identified expressed MST genes were higher than the average.

**Table 1:** Summary and analysis of EST database search results. Search results from all eight databases are summarized, including database size and number of ESTs showing significant similarity to each MST subfamily on BLASTX search for each taxon. For the five large databases (>100,000 ESTs), the percentage of subfamily ESTs as a proportion of total MST ESTs, the percentage of MST ESTs as a proportion of total ESTS, and the number of expressed loci is shown.

**Taxon**	**ESTdb size**	**ESTs with significant homology to the MST subfamily** **% subfamily ESTs as a proportion of total MST ESTs** **Number of expressed subfamily gene loci in EST database**							**Total MST ESTs ** **% MST ESTs** **Expressed loci**
		**STP**	**AZT**	**ERD6-like**	**pGlcT**	**INT**	**PLT**	**XyloseTP-like**	
***Marchantia*** (liverwort)	1,415	2							2 0.14%
***Physcomitrella ***(moss)	140,617	14 9.7% 3	54 37.5% 5	5 3.5% 2	54 37.5% 5	11 7.6% 2		6 4.2% 1	144 0.10% 18
***Selaginella ***(lycophyte)	1,046			1	1				2 0.19%
***Ceratopteris ***(fern)	5,085	1	1		1	1	1	1	6 0.12%
***Pinus ***(gymnosperm)	291,588	83 27.4% 21	8 2.6% 2	36 11.9% 10	64 21.1% 5	58 19.1% 14	41 13.5% 8	13 4.3% 2	303 0.10% 62
***Zea ***(monocot)	417,803	61 19.6% 15	51 16.3% 4	62 19.9% 9	41 13.1% 5	38 12.2% 3	47 15.1% 8	12 3.8% 2	312 0.07% 46
***Lycopersicon ***(eudicot – asterid)	189,735	27 29.0% 6	3 3.2% 1	33 35.5% 7	8 8.6% 3	4 4.3% 2	10 10.8% 3	8 8.6% 2	93 0.05% 24
***Arabidopsis ***(eudicot – rosid)	415,250	507 43.9% 10	41 3.5% 2	352 30.5% 18	71 6.1% 4	32 2.8% 4	111 9.6% 3	41 3.6% 3	1155 0.28% 44

### *Arabidopsis thaliana *EST database search

**Figure 2:**
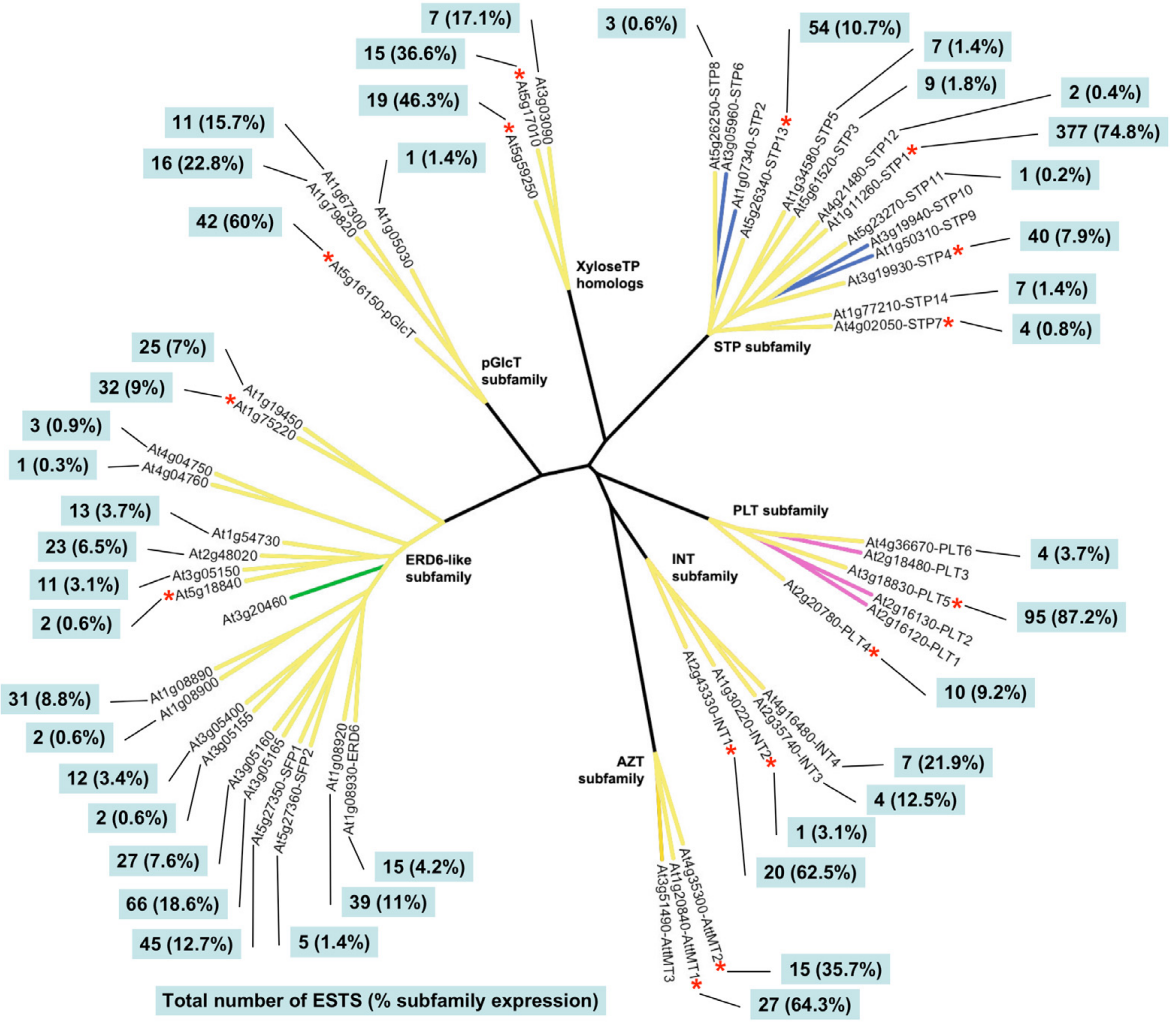
**Representation of MST genes in *Arabidopsis *EST database**. This tree is a radial representation of the maximum likelihood protein tree in Figure 1. Bootstrap values have been omitted and branch lengths have been modified to enhance visibility. Branches with yellow highlighting indicate the presence of ESTs in the *Arabidopsis thaliana *EST database of 415,250 ESTs. Callouts show the total number of ESTs with a best match to each indicated gene locus and the percentage of total subfamily expression levels. Red asterisks indicate genes with best match homologs present in at least one early lineage (*Marchantia*, *Physcomitrella*, *Selaginella*, *Ceratopteris*, or *Pinus*).

The proportion of known MST genes represented in the *Arabidopsis *EST database of 415,250 ESTs was 83% (44/53 genes) (Figure 2). Genes not represented in the database were *AtSTP2*, *-6*, *-10*, *-11*, AZT subfamily locus At3g51490, ERD6-like subfamily locus At3g20460, and *AtPLT1*, *-2*, and *-3*. Four subfamilies (STP, pGlcT, INT and PLT) showed a pattern in which one or a few genes had much higher representation in the EST database than any of the remaining expressed genes. In the STP subfamily, *AtSTP1 *had the highest representation in the EST database overall, with 377 ESTs expressed in a variety of tissues, stages and conditions. In the pGlcT subfamily, *AtpGlcT *exhibited a nearly 3-fold higher representation than the next most abundantly represented gene, as did *AtINT1 *in the INT subfamily. In the PLT subfamily, *AtPLT5 *had a 9- and 23-fold greater representation than *AtPLT4 *and *AtPLT6 *respectively. In the AZT, ERD6-like, and XyloseTP-like subfamilies, representation of gene subfamily members was more evenly distributed. Contig assembly (with a 95% overlap identity cutoff) of *Arabidopsis *ESTs revealed the presence of multiple contigs for most genes (data not shown). One or more gaps in sequence distinguished the contigs in these cases, indicating the presence of alternative splicing variants. Details regarding the EST records, BLASTX results, and contigs are contained in Additional file 11.

### Small EST database searches

**Figure 3:**
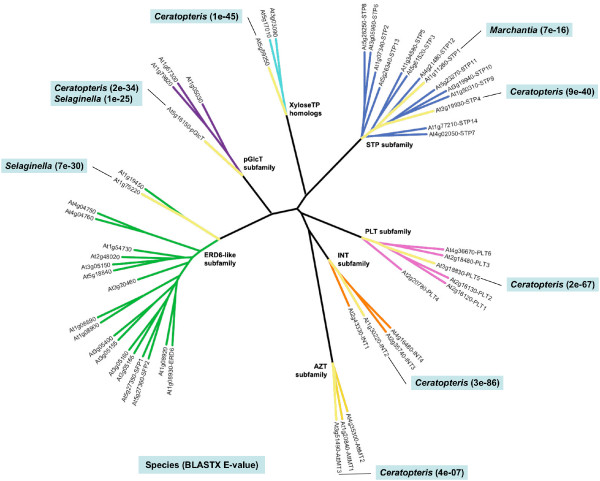
**Expressed MST loci in small EST databases**. Radial ML tree of *Arabidopsis *MST proteins with branches highlighted in yellow to denote the presence of ESTs in one of the small EST databases with a best match to that particular *Arabidopsis *gene. Callouts label the species name and e-value of the match.

Expressed MST genes were identified in all seven subfamilies from at least one of the three small EST databases of the early divergent plant lineages (Figure 3). Specifically, two ESTs were identified in *Marchantia *(STP subfamily), two in *Selaginella *(pGlcT and ERD6-like subfamilies) and six ESTs in *Ceratopteris *(all subfamilies except ERD6-like). Details regarding the EST records and BLASTX results are contained in Additional file 12.

### *Physcomitrella patens *EST database searches

**Figure 4:**
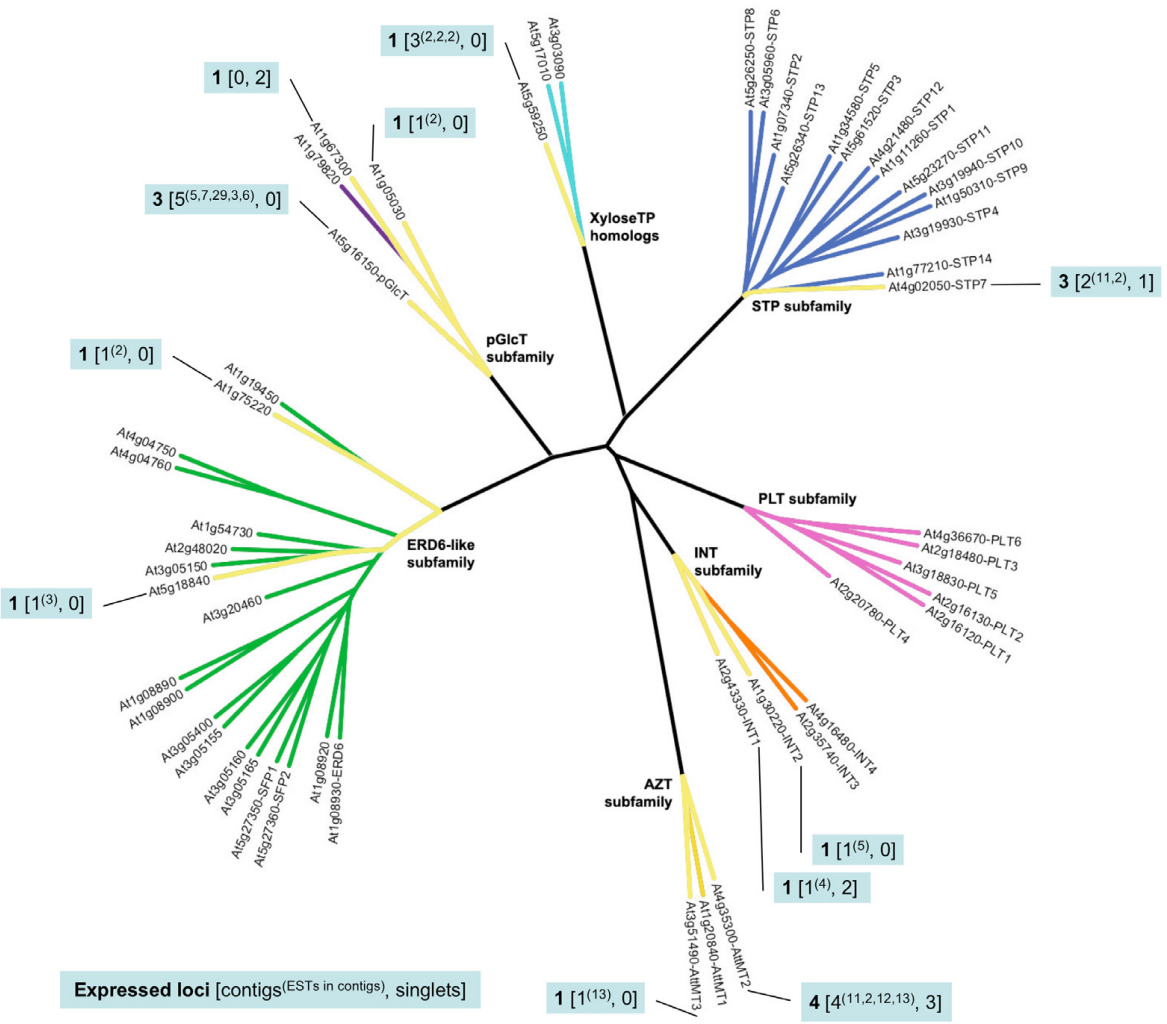
**Expressed MST loci in the *Physcomitrella patens *EST database**. Radial ML tree of *Arabidopsis *MST proteins with branches highlighted in yellow to indicate the presence of EST contigs or singlets in the *Physcomitrella patens *EST databases with a best match to the indicated *Arabidopsis *gene. Callouts indicate **Number of inferred expressed loci **[number of EST contigs^(# of ESTs in each contig)^, number of singlets].

Two *Physcomitrella *databases were searched (*Physcomitrella patens *and *Physcomitrella patens *subsp. *patens*) for a combined 140,617 ESTs. Contig assembly and analysis revealed a minimum of 18 expressed loci across the seven MST subfamilies (Figure 4). No ESTs showing similarity to the PLT subfamily were identified. Details regarding the EST records, BLASTX results, and contigs are contained in Additional file 13.

### *Pinus taeda *EST database search

**Figure 5:**
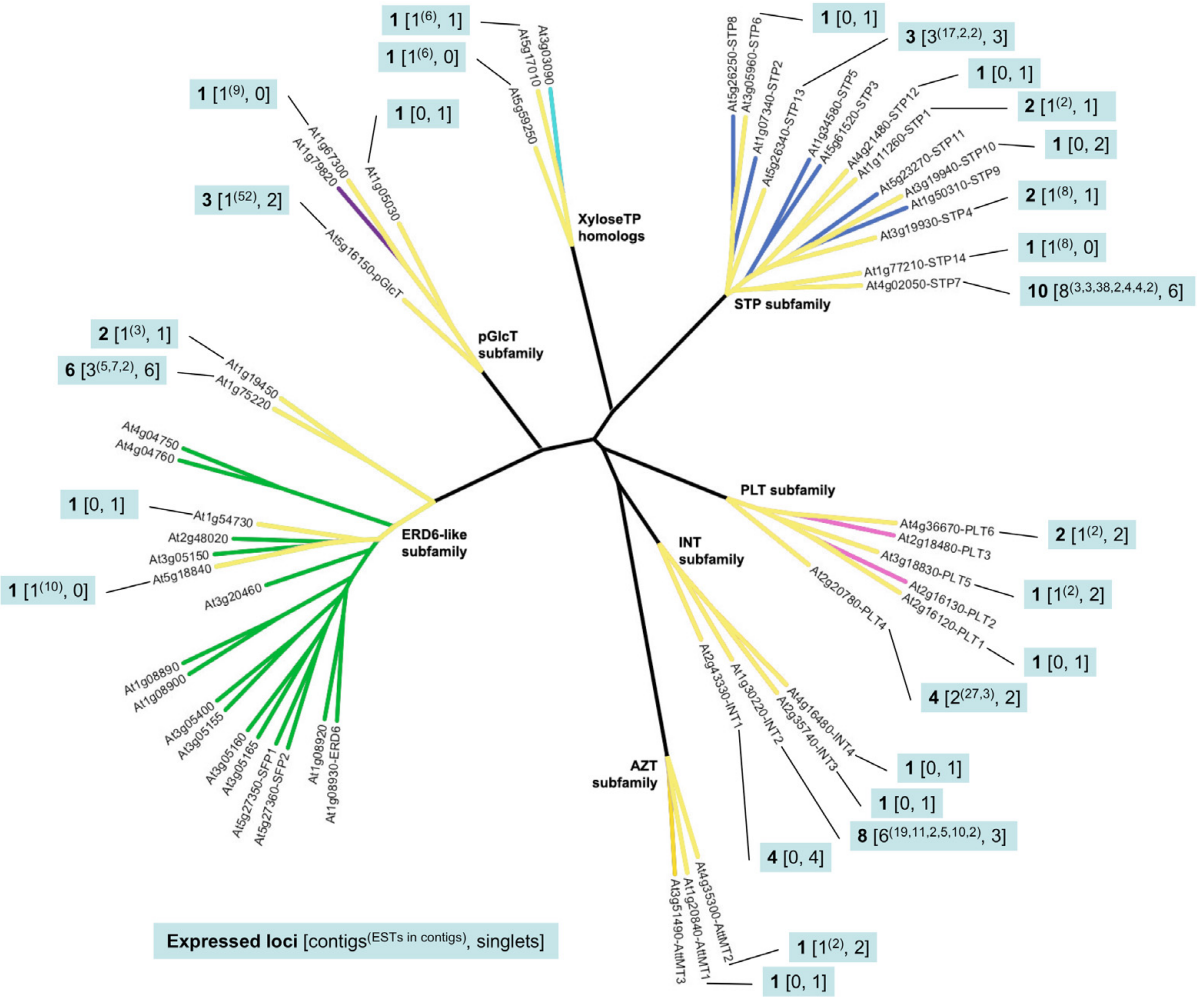
**Expressed MST loci in the *Pinus *taeda EST database**. Radial ML tree of *Arabidopsis *MST proteins with branches highlighted in yellow to indicate the presence of EST contigs or singlets in the *Pinus taeda *EST database with a best match to the indicated *Arabidopsis *gene. Callouts indicate **Number of inferred expressed loci **[number of EST contigs^(# of ESTs in each contig)^, number of singlets].

Contig assembly and analysis revealed a minimum of 62 expressed loci across the seven MST subfamilies (Figure 5). Details regarding the EST records, BLASTX results, and contigs are contained in Additional file 14. Of note is that there are several cases of subfamily expansion due to multiple gene duplications along a single gene lineage. For example, in the STP subfamily, ten expressed loci show highest similarity to the *ATSTP7 *gene, suggesting multiple rounds of gene duplication (probably tandem) arising from the ancestral *STP7 *ortholog in *Pinus taeda*. Similar gene duplication clusters are present in the ERD6-like, PLT, and INT subfamilies, resulting in large expansions of these subfamilies.

### *Zea mays *EST database search

**Figure 6:**
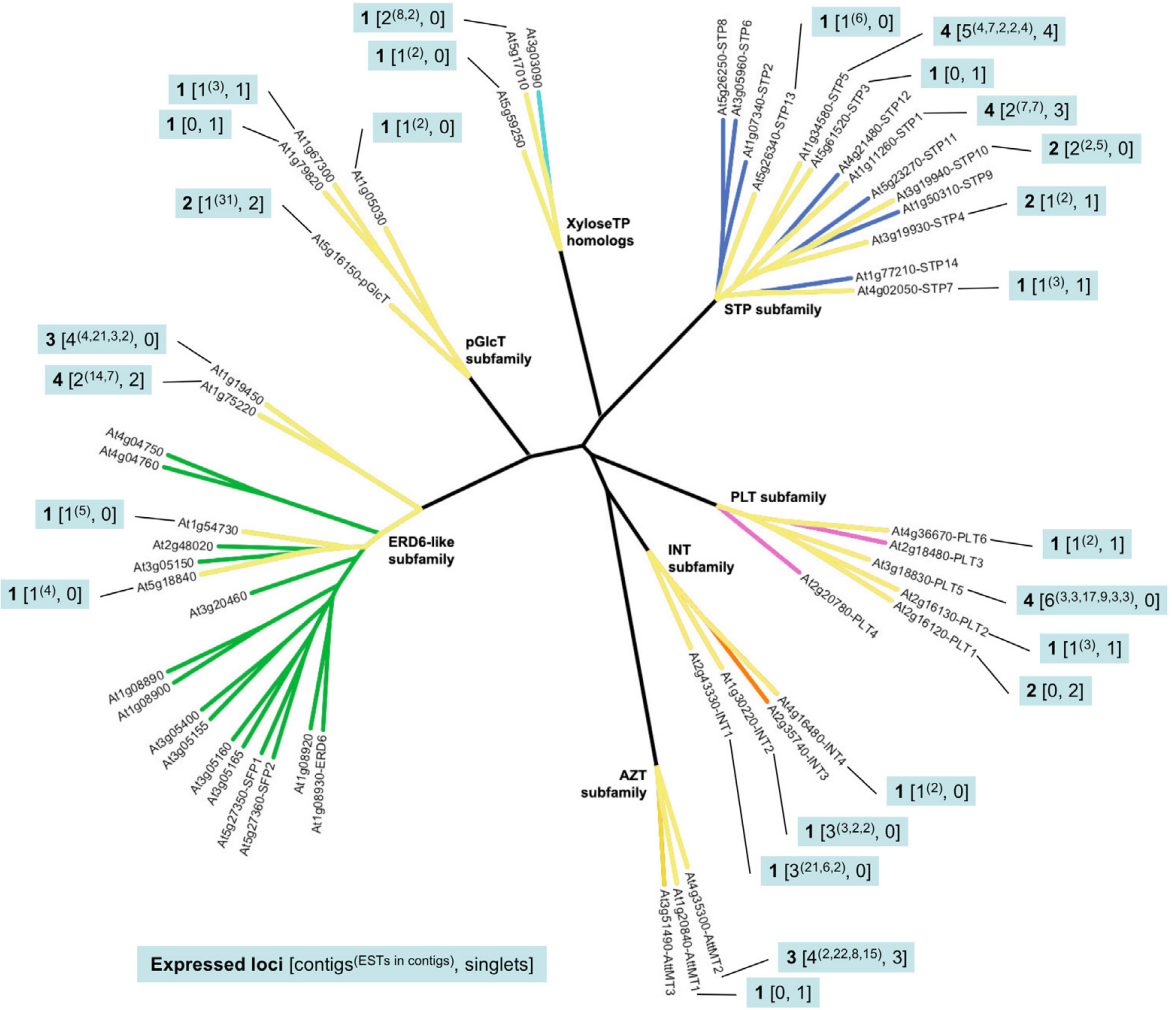
**Expressed MST loci in the *Zea mays *EST database**. Radial ML tree of *Arabidopsis *MST proteins with branches highlighted in yellow to indicate the presence of EST contigs or singlets in the *Zea mays *EST database with a best match to the indicated *Arabidopsis *gene. Callouts indicate **Number of inferred expressed loci **[number of EST contigs^(# of ESTs in each contig)^, number of singlets].

Contig assembly and analysis revealed a minimum of 46 expressed loci across the seven MST subfamilies (Figure 6). Details regarding the EST records, BLASTX results, and contigs are contained in Additional file 15. As in *Pinus*, there is evidence of subfamily expansion due to multiple gene duplications arising from individual ancestral orthologs. However, the number of duplications in *Zea *(as seen in this EST data) does not exceed four in any single case.

### *Lycopersicon esculentum *EST database search

**Figure 7:**
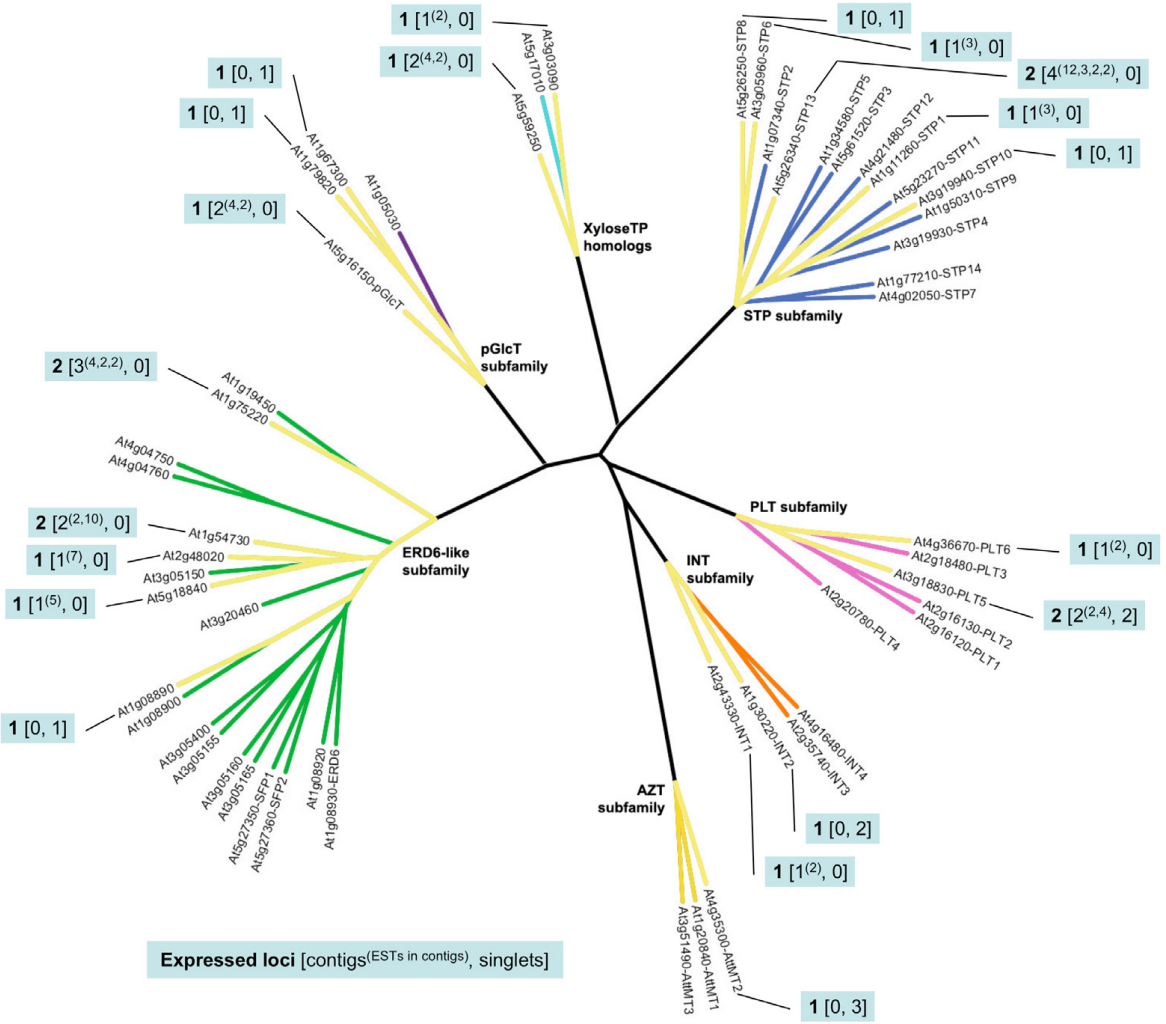
**Expressed MST loci in the *Lycopersicon esculentum *EST database**. Radial ML tree of *Arabidopsis *MST proteins with branches highlighted in yellow to indicate the presence of EST contigs or singlets in the *Lycopersicon esculentum *EST database with a best match to the indicated *Arabidopsis *gene. Callouts indicate **Number of inferred expressed loci **[number of EST contigs^(# of ESTs in each contig)^, number of singlets].

Contig assembly and analysis revealed a minimum of 24 expressed loci across the seven MST subfamilies (Figure 7). Details regarding the EST records, BLASTX results, and contigs are contained in Additional file 16. The remarkable feature of the *Lycopersicon *EST database search is the low number of MST ESTs present in the relatively large EST database with a concordant low number of expressed loci. A likely reason for this is ascertainment bias in the cDNA libraries from which the ESTs were derived. However, a review of the tissue and organ types, developmental stages and growth conditions represented in the EST database reveals them to be varied, including callus, leaf, root, shoot, flower, and fruit at various developmental stages and/or conditions.

## Discussion

### The seven subfamilies of MST genes are ancient

The results of our EST database searches confirm that all of the *Arabidopsis *MST subfamilies but one, the PLT (polyol transporter) subfamily, are found in the EST database of the early divergent moss lineage (*Physcomitrella patens*), dating back >410 million years ago. Because polyols are known osmoprotectants in stress conditions and many bryophytes are known to leach sugars and polyols after freeze-thaw events in cold temperature environments [55], it seems likely that bryophytes possess polyol transporters. *Physcomitrella patens*, specifically, has been shown to be drought, salt and freezing-tolerant [56,57]. The largest *Physcomitrella *EST database searched (120,702 ESTs) was constructed from auxin and cytokinin-treated gametophytes and gametophytes with no hormone treatment [49]. Abscisic acid (ABA) is known to induce stress-related genes in plants. However, of the remaining *Physcomitrella *ESTs searched (19,914 ESTs), only 2,492 ESTs were derived from ABA-treated gametophytes (1.8% of total ESTs in the clustered *Physcomitrella *EST database). It may be that expression of PLT genes in *Physcomitrella patens *is induced by stress and that stress-induced genes are significantly underrepresented in the clustered *Physcomitrella *EST database, resulting in the absence of many stress-induced genes in the database. However, absence of this subfamily of transporters in this species may also be real. The earliest divergent lineage in which we found ESTs from the PLT subfamily is the fern lineage *(Ceratopteris richardii)*, which diverged from its common ancestor with the seed plants approximately 400 million years ago. We found no expressed PLT genes in the EST database of the lycophyte *Selaginella lepidophylla*, but absence of this subfamily from this EST database may be an artifact of its small size (1,046 ESTs).

### MST gene family size varies across lineages

The total number of expressed MST loci in each species varied from 18 to 64 in the five lineages with large EST databases. Not surprisingly, the smallest number of expressed MST loci (18) was found in the early divergent moss, *Physcomitrella patens*. The largest number of expressed MST loci (64) was found in the gymnosperm *Pinus taeda*, with the angiosperms *Zea mays *and *Arabidopsis thaliana *having similar numbers of expressed MST loci (46 and 44 respectively). Interestingly, the number of expressed MST loci in the *Lycopersicon esculentum *EST database, at 24, is almost half as large as the number of expressed loci in the *Zea *and *Arabidopsis *databases, and not much larger than the number of loci expressed in the nonvascular moss. Possible explanations include a significant bias in the *Lycopersicon *database or a paucity of large and/or small-scale gene duplication events in the *Lycopersicon *lineage, resulting in small gene family sizes compared to lineages in which gene duplication events have been more numerous. Given that the cDNA libraries from which the *Lycopersicon *ESTs were derived appear to be as varied in tissue/organ type and developmental stage as the other large EST databases analyzed, it appears likely that bias in the database does not completely explain the low numbers of expressed MSTs. Comparison of sequenced segments of the tomato genome with the *Arabidopsis *genome reveals multiple large-scale genome duplications are present in *Arabidopsis *[58] but not *Lycopersicon*, suggesting that the low numbers of expressed MST loci in tomato may be a result, at least in part, of fewer large-scale genome duplication events in its evolutionary history. A lower level of tandem gene duplication events (at least in the MST gene family) may also be a factor in the low number of expressed MST loci.

### MST subfamily expression levels differ across lineages

The relative expression level of most subfamilies, inferred from these EST data, appears to have changed across the major land plant lineages, with the AZT and pGlcT gene subfamilies having much greater proportional expression in the early moss lineage (37.5% each for AZT and pGlcT) than in the gymnosperm (2.6% and 21.1%), monocot (16.3% and 13.1%) and eudicot lineages (averages of 3.4% and 7.4%) (Table 1). Conversely, proportional expression levels of the STP subfamily are greatly increased in the gymnosperm and angiosperm lineages (an average of 30%) as compared to the moss (9.7%). Relative expression levels of the ERD6-like subfamily also show an increase from the lowest levels in the moss (3.5%) through the gymnosperm (11.9%), monocot (19.9%) and eudicot lineages (average of 33%). It is possible that these differences in relative expression levels for each subfamily reflect bias in the cDNA libraries from which the ESTs in each database were derived. However, since each of the large databases contains large numbers of ESTs derived from whole plant tissue and major organ tissues under different conditions, it is likely that these data do reflect some real differences in relative subfamily expression. In the absence of functional characterization and expression studies for genes in each of the subfamilies, it is premature to speculate about the functional significance of these relative expression differences. However, given that monosaccharide transporters are associated with a variety of sink tissues, co-expressed cell wall invertases and phloem unloading in the vascular plants [21], we would predict an increase in size and complexity of this gene family. Increased expression and expansion of the STP, ERD6-like, INT and PLT subfamilies in the vascular plants, therefore, may reflect the evolution of structural and physiological complexity associated with vascularity.

In addition, the proportion of MST ESTs identified in each of the databases varied between 0.05% in *Lycopersicon *and 0.28% in *Arabidopsis *(Table 1) with an average of 0.13%. The small EST databases showed percentages of MST ESTs ranging from 0.12–0.19%. A potential source of differences in relative proportion of MST ESTs identified in each EST database is a bias in the sequences from which the profile HMMs were constructed. However, this seems unlikely, given that there was one gymnosperm, many monocot and two *Lycopersicon *MST sequences included in the profile HMM sequence set and none for the very early divergent liverwort, moss, and lycophyte lineages, but percentages of MST ESTs identified in the liverwort, moss and lycophyte databases are higher (0.10–0.19%) than in *Lycopersicon *and *Zea*. It seems noteworthy that the percentage of MST ESTs in the *Arabidopsis *database is substantially higher (0.28%) than in any of the other species (0.05–0.19%).

### MST gene expression in *Arabidopsis*

In this large database of 415,250 ESTs, derived from multiple plant stages, tissue types and treatments, we found ESTs representing 44 of the 53 known MST genes (83%). Of the nine genes for which we found no ESTs, the function and expression of three are known. Interestingly, these three genes (*AtSTP2*, *-6*, and *-9*) are expressed in developing pollen only (the haploid male gametophyte). One other gene known to be expressed in pollen only is *AtSTP11*, for which we found one EST derived from a mixture of silique and flower tissue. Absence of the *AtSTP2*, *-6 *and *-9 *genes from the *Arabidopsis *EST database, then, seems likely due to the paucity of transcripts from pollen development in the database. This invites speculation that some or all of the six other genes missing from the database may also be expressed in gametophyte tissue only. An evaluation of microarray gene expression data contained on the Weigel World website (AtGenExpress Development) [59,60] reveals that of the six remaining missing MST genes, three have expression profiles consistent with pollen-specific expression (*AtSTP10*, *AtPLT1*, and *AttMT3*), with one gene not present on the array (*AtPLT2*). An examination of the adaptive significance of *Arabidopsis *MST gene expression in relation to rates of sequence evolution is currently underway in our laboratory (data unpublished).

Overall patterns of individual MST gene EST database representation in some subfamilies reveal that one, or a few, genes have significantly greater representation than the others. For example, in the STP subfamily, *AtSTP1 *(74.8%), *AtSTP13 *(10.7%) and *AtSTP4 *(7.9%) make up 93.4% of 10 represented STP genes. In the PLT subfamily, the *AtPLT5 *gene makes up 87.2% of total subfamily representation. In the greatly expanded ERD6-like subfamily, however, gene representation is apportioned more equitably with most genes ranging between 3% and 10% of the total EST database representation. The ERD6-like gene with the highest representation (18.6%) is *AtSFP1*. The large expansion of this subfamily is due to tandem duplications involving four clusters of genes, with no other *Arabidopsis *MST subfamily showing this high level of tandem duplication. Most of the highly represented ERD6-like genes are members of these tandem arrays.

High representation of a specific gene in the combined *Arabidopsis *EST database may be the result of high expression (transcript levels) in one or more organs or lower expression in many different organs (broad expression). However, many cDNA libraries from which ESTs are derived are normalized, eliminating redundant transcripts of genes with high or broad expression and increasing the relative proportion of transcripts from genes with low or narrow expression. Thus, there may be genes with relatively low representation in the EST database that are, in fact, more broadly or highly expressed than indicated by their relative presence in the database.

### The MST subfamilies have lineage-specific expansion patterns

**Figure 8:**
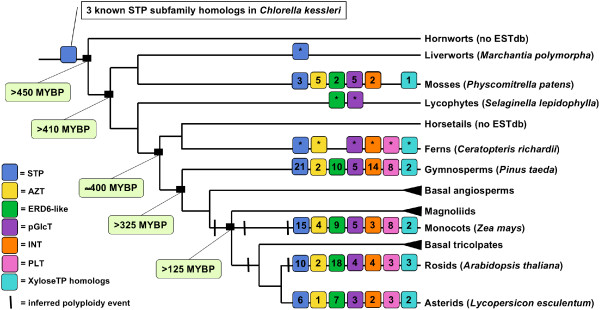
**Lineage divergence times, inferred polyploidy events and number of MST subfamily loci inferred from EST data, presented in phylogenetic context**. Phylogeny showing hypothesized relationships among major land plant lineages [70], approximate divergence times [70], with vertical bars indicating inferred polyploidy events [61]. Colored squares indicate presence of one or more subfamily homologs within a lineage, numbers within squares indicate the number of expressed loci, and *'s indicate EST databases with too few ESTs to infer numbers of expressed loci. Species names on selected lineages indicate EST databases searched in this study.

**Figure 9:**
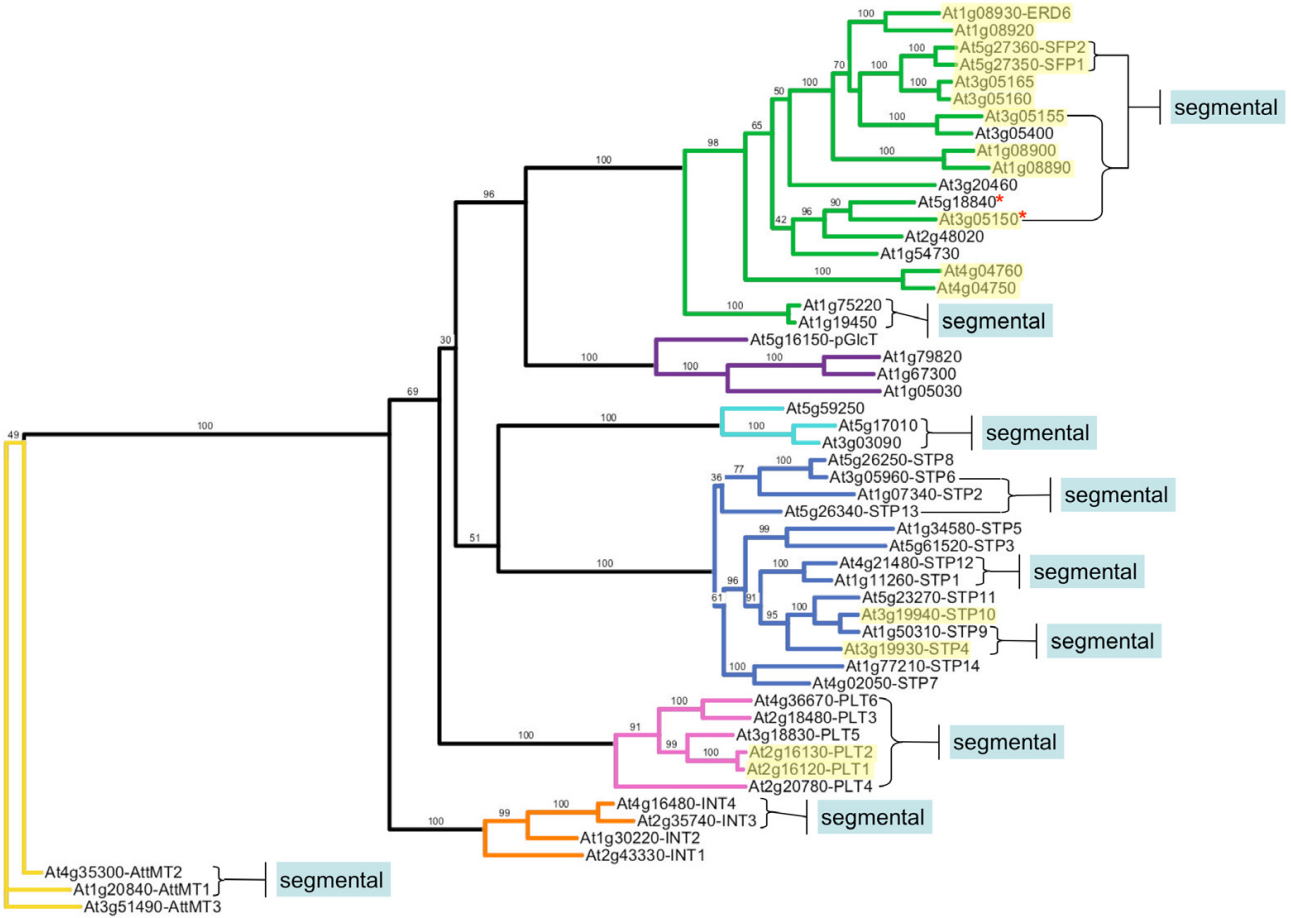
***Arabidopsis *MST gene duplication events in phylogenetic context**. Maximum likelihood phylogeny of *Arabidopsis *MST protein sequences with segmental duplication events indicated by callouts and tandem duplications indicated by yellow highlighting. Red * symbols indicate two genes with high similarity likely duplicated by segmental duplication unrecognized on the TIGR Arabidopsis Genome Annotation database.

Our data present clear evidence that the MST subfamilies have experienced lineage-specific expansions across the land plant family tree (Table 1 and Figure 8). In the earliest lineage with a large EST database, *Physcomitrella patens*, each subfamily is comparatively small (between one and five expressed loci) (Figure 4) relative to the gymnosperm and angiosperm lineages. Its two largest subfamilies, AZT and pGlcT, each have five expressed loci. In the AZT subfamily, four loci appear to be the result of repeated duplications arising in the ancestral *AttMT2 *gene lineage. In the pGlcT subfamily, three loci appear to be the result of duplications arising in the ancestral *AtpGlcT *gene lineage. The gymnosperm *Pinus taeda *has four expanded subfamilies, STP, ERD6-like, INT, and PLT (Figure 5). Almost half of the STP subfamily expansion in *Pinus taeda *is the result of ten duplications in the ancestral *AtSTP7 *gene lineage. In the INT subfamily, eight duplications in the ancestral *AtINT2 *gene lineage and four gene duplications in the ancestral *AtINT1 *gene lineage result in 86% of expressed loci. The monocot angiosperm *Zea mays *has three expanded subfamilies, STP, ERD6-like and PLT (Figure 6). In the STP subfamily, two ancestral genes orthologous to *AtSTP5 *and *AtSTP1 *each experienced four gene duplications, resulting in more than half of the *Zea mays *STP genes. The eudicot angiosperm *Lycopersicon esculentum *has two slightly expanded subfamilies, STP and ERD6-like, but there are no instances where more than two expressed loci appear to have been duplicated from one ancestral gene lineage (Figure 7). In *Arabidopsis *the STP and ERD6-like subfamilies are large. Mapping of duplication events on the phylogeny (Figure 9) reveals three segmental duplications and one tandem duplication discernible in the STP subfamily. Two segmental duplications (one involving two genes) and six apparent tandem duplications have resulted in the large expansion of the ERD6-like subfamily. Differences in subfamily size among the monocot and dicot rosid and asterid lineages are likely correlated to the number of whole genome duplication events in their evolutionary histories (Figure 8). A recent analysis of EST data and completed genome sequence for 14 model plant species has inferred three polyploidy events in the monocot lineage, three in the dicot rosid lineage but only two in the dicot asterid lineage [61] (Figure 8). This likely explains, at least in part, the relatively lower number of expressed loci in the *Lycopersicon *lineage.

### Is broad *Arabidopsis *MST gene expression correlated with the presence of orthologs in early lineages?

High gene expression levels have been associated with slow sequence evolution rates in yeast [16] and eukaryotes [17]. In addition, a study in vertebrates [18] and another in mammals [19] present strong evidence that genes with broad expression are under stronger purifying selection than genes with tissue-specific expression. Two models have been proposed to explain this: (1) A protein with broad expression would be exposed to more diverse biochemical environments and, hence, would be under stronger purifying selection [18]. (2) A broadly expressed protein with a deleterious mutation would most likely have a greater impact on organismal fitness than a protein with narrow, tissue-specific expression and would, thus, experience greater purifying selection [19].

We performed a simple analysis of the relationship between *Arabidopsis *MST genes with relatively high EST database representation within subfamily and the presence of best match MST homologs in early lineages to explore patterns that might be consistent with these studies. We assumed that, because the combined *Arabidopsis *EST database is derived from many different tissues, developmental stages and growth conditions, an *Arabidopsis *gene with high representation in the database, relative to other members of its subfamily, is likely a gene with high and/or broad expression, and a gene with low representation is likely a gene with low and/or narrow expression. We evaluated this assumption with an analysis of the AtGenExpress *Arabidopsis *developmental microarray gene expression data [60]. An expressed gene in an early divergent lineage with a best match to an *Arabidopsis *gene was used an as indication that the *Arabidopsis *gene is evolving relatively slowly under strong purifying selection.

**Figure 10:**
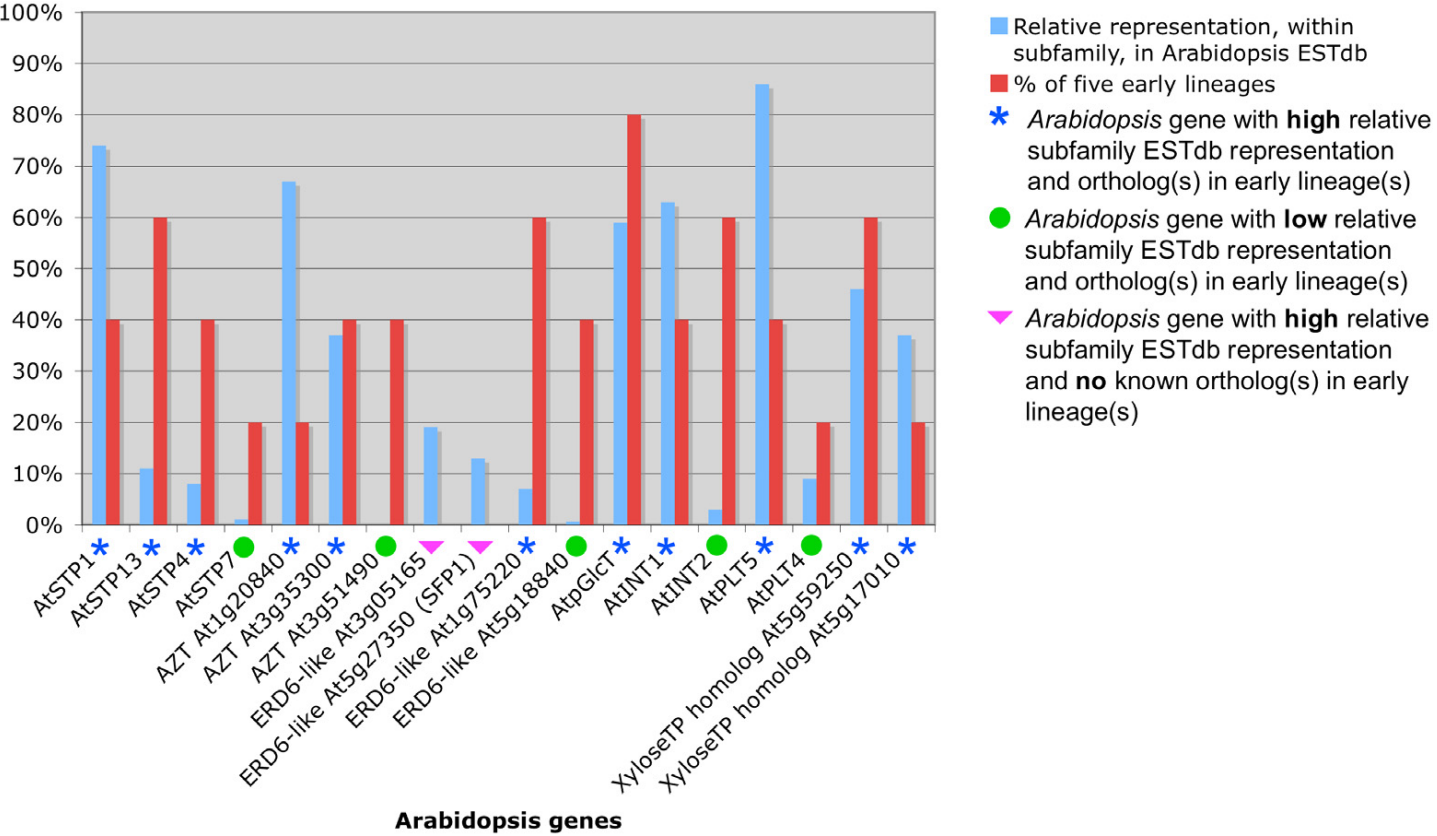
**Correlation between high EST database representation of Arabidopsis MST genes and the presence of best match orthologs in one or more early land plant lineages**. A bar chart showing the relationship between percent relative subfamily EST database representation and presence of best match orthologs in one or more representatives of five early land plant lineages (*Marchantia polymorpha*, *Physcomitrella patens*, *Selaginella lepidophylla*, *Ceratopteris richardii*, and *Pinus taeda*). *Arabidopsis *genes with high relative subfamily representation and/or best match homologs in the early lineages are included in the chart.

Of 13 MST genes with relatively high subfamily representation, 11 have best match homologs in one or more of the five early divergent land plants included in our study (liverwort, moss, lycophyte, fern, or gymnosperm) (Figure 10). Of these, the expression of three (*AtSTP1*, *AtSTP4*, and *AtPLT5*) has been characterized and can be considered broad, i.e., expressed in multiple organs and developmental stages. *AtSTP1 *is expressed in germinating seeds and seedlings, concentrated in the root, and also in guard cells [26,27]. *STP4 *is expressed in classic sink tissues such as root tips, pollen and anthers and in tissues damaged by environmental stresses [28]. *PLT5 *is expressed most strongly in roots but also in vascular tissue of leaves and in floral organs [34]. Microarray expression profiles from the developmental AtGenExpress dataset reveal that all of the MST genes we identified with high representation in the EST database (with the exception of At3g51490 which is not present on the array) have profiles consistent with broad expression across three or more major plant structures (root, stem, leaf, flower, and/or seed). The two ERD6-like subfamily genes with high EST database representation that lack the presence of orthologs in early lineages also have microarray profiles consistent with broad expression. These two genes are both members of a large subclade of tandemly duplicated genes in the ERD6-like subfamily and may have undergone substantial divergence from an ancestral gene present in an early lineage. Of the five MST genes with relatively low, or no, representation in the database and best match homologs in early lineages, three (*AtSTP7*, *At5g18840*, and *AtINT2*) have microarray expression profiles consistent with broad expression and two (*At3g51490 *and *AtPLT4*) appear to have limited expression, including expression in pollen. The broadly expressed genes may have low EST database representation as a result of normalized cDNA libraries and/or because they have low expression levels (making our analysis of the correlation between high EST database representation and the presence of orthologs in early lineages conservative). The genes expressed in pollen may be conserved descendants of ancestral MST genes present in the earliest land plants, and thus have orthologs present in the extant members of these early lineages.

### Implications for the role of MST gene duplication in early land plant evolution

Previous comparison of a large collection of *Physcomitrella *ESTs with protein sequences from the *Arabidopsis *genome [49] concluded that the *Physcomitrella *gametophyte transcriptome was 'quite similar' to the *Arabidopsis *genome, supporting the hypothesis that genes expressed in the sporophytes of higher plants were 'recruited' from genes expressed in the gametophytes of early land plant ancestors, resulting in a set of 'shared genes' in the two lineages. However, if gene duplication events are prevalent in plants and the most common fate of retained gene duplicates is a partitioning of gene expression, a large number of *Arabidopsis *genes that show similarity to genes expressed in the *Physcomitrella *gametophyte are likely to be paralogs, and not direct descendants of the same genes expressed in the gametophyte of a common ancestor. A recent genome-scale comparison of the *Arabidopsis *male gametophyte (pollen) transcriptome with the sporophyte transcriptome revealed a 61% overlap in expression of 992 pollen-expressed mRNAs, with nearly 40% of these showing pollen-specificity [62]. Our analysis of MST gene transcripts represented in the *Arabidopsis *EST database and the AtGenExpress microarray expression data suggests that at least seven of the 43 MSTs may be gametophyte-specific.

Our comparison of MST genes in the transcriptomes of *Physcomitrella patens*, *Pinus taeda*, *Zea mays *and *Lycopersicon esculentum *with the known MST gene family in *Arabidopsis thaliana *also allowed us to identify significant differences in size between the STP and ERD6-like subfamilies in the nonvascular moss lineage and the vascular gymnosperm and angiosperm lineages, suggesting that expansion of these subfamilies is potentially related to the evolution of the vascular sporophyte. Hypotheses about the origin and evolution of the complex vascular sporophyte must include explicit ideas about the role of gene duplication and divergence in the expression of genes in the separate haploid and diploid phases of the plant life cycle. The significance of MST gene duplication on plant life cycle evolution remains to be revealed through appropriate empirical studies.

## Conclusion

The subfamilies of MST genes present in *Arabidopsis *are ancient, with six of the seven subfamilies (STP, AZT, ERD6-like, pGlcT, INT and XyloseTP homologs) found in the moss lineage, which diverged from its common ancestor with the vascular plants >410 million years ago. Among the EST databases that we searched, the earliest lineage in which a homolog of the PLT subfamily was identified was the fern lineage, which diverged from its common ancestor with the vascular plants about 400 million years ago. The PLT subfamily may be present in earlier lineages but developmental or environmental conditions under which these transporters are expressed may not be present in the EST databases.

The MST subfamilies also show lineage-specific subfamily expansion patterns. Subfamily expansion in the vascular plants often appears to be due to multiple gene duplications arising from a single ancestral gene (likely tandem duplications) within a subfamily. In *Arabidopsis*, the large expansion of the ERD6-like subfamily is due to four clusters of tandem duplications involving 68% of subfamily genes.

Relative subfamily expression levels, inferred from EST data, vary across lineages. There is greater expression of the STP and ERD6-like subfamilies in the gymnosperm and flowering plant lineages, with relatively high expression of the AZT and pGlcT subfamilies in the nonvascular moss lineage. These differences may reflect increased roles of the STP and ERD6-like subfamilies in the long-distance transport of sugars in vascular plants.

EST database representation of individual *Arabidopsis *genes indicates that one or a few genes within a subfamily often have much higher representation than others. These patterns are consistent with models of gene duplicate divergence in which a gene duplicate assumes a portion of the function and/or expression of a progenitor gene having broad function and/or expression. However, the largest *Arabidopsis *subfamily, the ERD6-like subfamily, does not fit this pattern, with a large number of tandem gene duplicates having more equitable EST database representation.

Correlation of *Arabidopsis *genes with high EST database representation with the presence of orthologs in early lineages reveals that 11 of 13 highly represented genes (85%) have best match homologs. This is consistent with hypotheses that genes with high and/or broad expression are more conserved than genes with narrow expression. Of the five *Arabidopsis *genes with little or no representation in the database that also had best match homologs in early lineages, three had microarray expression profiles consistent with broad expression and two had more narrow expression patterns, including expression in pollen (male gametophyte). These genes expressed in the haploid phase may be conserved descendants of ancestral genes expressed in the dominant or independent haploid phase of early land plants and, in the absence of another allele, are likely to be under strong purifying selection.

## Methods

### Phylogenetic analysis and gene mapping

Phylogenetic analysis of the 53 *Arabidopsis *MST protein sequences using maximum likelihood and maximum parsimony was performed. A maximum likelihood tree with 100 bootstrap replicates was constructed with the PHYML program [63] using the JTT amino acid substitution model, a discrete gamma model with four categories and an estimated shape parameter of 1.385. A consensus maximum parsimony tree was constructed with PAUP*, version 4.0 beta 10, with 10,000 bootstrap replicates using the tree-bisection-reconnection branch-swapping heuristic search algorithm. All 53 *Arabidopsis *MST genes were mapped onto the five *Arabidopsis *chromosomes using the TIGR locus tags and the TAIR Chromosome Map Tool [64]. Segmental chromosome duplications were identified using the TIGR *Arabidopsis thaliana *Genome Annotation database [65].

### Profile hidden Markov models and consensus sequences

One hundred twenty MST genes from across the green plant lineage were assembled by searching the pfam database for all full- or nearly full-length MST genes in the viridiplantae (62 sequences), combining these with the 53 *Arabidopsis *MST genes reported on the TAIR *Arabidopsis *Monosaccharide Transporter(-like) Gene Family website [41], three green algal (*Chlorella kessleri*) MST genes (accession #'s P15686, Q39524, Q39525), and two partial fern (*Ceratopteris richardii*) MST genes (accession #'s DQ866147 and DQ866148). The fern genes were amplified by degenerate PCR in our laboratory and translated based on BLASTX searches. The 120 protein sequences were aligned with ClustalW [66] and a neighbor-joining phylogenetic tree was produced with PAUP* 4.0 Beta 10 [67] to determine the subfamily identity of each MST sequence. Each subfamily group of protein sequences was then realigned with ClustalW and a profile HMM and consensus sequence produced with the HMMER software package [68], version 2.3.2.

### EST database searches

Nine EST databases (*Marchantia polymorpha*, *Physcomitrella patens*, *Physcomitrella patens *subsp. *patens*, *Selaginella lepidophylla*, *Ceratopteris richardii*, *Pinus taeda*, *Zea mays*, *Arabidopsis thaliana*, and *Lycopersicon esculentum*) were downloaded from the NCBI website and installed on our local server. Each database was searched using the *estwisedb *component of the Wise2 package [53], version 2.1.20, and the profile HMM-based consensus sequence for each MST subfamily. For the large EST databases, custom Perl scripts were written to automate the retrieval of FASTA files of EST sequences with significant e-values (< 1e-10) and to perform BLASTX searches against the *Arabidopsis *refseq database to identify the best match *Arabidopsis *gene for each EST sequence. Alignments of each translated EST sequence with its best match *Arabidopsis *gene were reviewed to confirm identity of each EST as a member of one of the seven MST subfamilies. The accession number for each positive EST sequence, locus tag for best match *Arabidopsis *MST gene, BLASTX e-value and cDNA library description were recorded in Excel spreadsheets for each EST database (Additional files 11, 12, 13, 14, 15, 16).

### Contig assembly and analysis

EST sequences showing a best match to the same *Arabidopsis *MST gene were assembled into contigs with the CAP3 program [69], using default parameters (75% overlap identity cutoff) for all species except *Arabidopsis*. For *Arabidopsis *we used a 95% overlap identity cutoff to exclude ESTs with significant sequencing errors and to combine ESTs from different alternative splicing isoforms into separate contigs. Expressed loci in each of the large non-Arabidopsis EST databases were determined by comparison of BLASTX alignments of EST contigs and singlets with their best match *Arabidopsis *MST protein. Separate EST contigs or singlets with significant overlap (>50 amino acids) and a best match to the same *Arabidopsis *MST gene but with different amino acid sequence were deemed to be different loci.

## Authors' contributions

DAJ conceived of the study, participated in the design of the study, created the hidden Markov models, performed the EST database searches, analyzed the data, and wrote the manuscript. MAT participated in the design of the study, the analysis of the data and in substantive revision of the manuscript. JPH participated in the concept of the study and in substantive revision of the manuscript. All authors read and approved the final manuscript.

## Supplementary Material

Additional File 1 Chromosome map of all 53 *AtMST *gene loci. All 53 MST loci were mapped on the five *Arabidopsis *chromosomes using the Chromosome Map Tool on the TAIR website. Arrows show segmental genome duplications. Red *'s indicate two genes likely involved in a segmental duplication not detected in the TIGR genome annotation database. Click here for file

Additional File 2 Pfam MST genes in viridiplantae clade used for subfamily profile HMM construction. Microsoft Word file listing all full- or nearly full-length monosaccharide transporter genes collected from the pfam database for all taxa except *Arabidopsis*, with SwissProt ID number, taxon and gene description listed. Click here for file

Additional File 3 Profile HMMs for each MST subfamily [STP, AZT, ERD6-like, pGlcT, INT, PLT, XyloseTP homologs]. Text file output from the *hmmbuild *component of the HMMer software package. Click here for file

Additional File 4 Profile HMMs for each MST subfamily [STP, AZT, ERD6-like, pGlcT, INT, PLT, XyloseTP homologs]. Text file output from the *hmmbuild *component of the HMMer software package. Click here for file

Additional File 5 Profile HMMs for each MST subfamily [STP, AZT, ERD6-like, pGlcT, INT, PLT, XyloseTP homologs]. Text file output from the *hmmbuild *component of the HMMer software package. Click here for file

Additional File 6 Profile HMMs for each MST subfamily [STP, AZT, ERD6-like, pGlcT, INT, PLT, XyloseTP homologs]. Text file output from the *hmmbuild *component of the HMMer software package. Click here for file

Additional File 7 Profile HMMs for each MST subfamily [STP, AZT, ERD6-like, pGlcT, INT, PLT, XyloseTP homologs]. Text file output from the *hmmbuild *component of the HMMer software package. Click here for file

Additional File 8 Profile HMMs for each MST subfamily [STP, AZT, ERD6-like, pGlcT, INT, PLT, XyloseTP homologs]. Text file output from the *hmmbuild *component of the HMMer software package. Click here for file

Additional File 9 Profile HMMs for each MST subfamily [STP, AZT, ERD6-like, pGlcT, INT, PLT, XyloseTP homologs]. Text file output from the *hmmbuild *component of the HMMer software package. Click here for file

Additional File 10 Alignment of MST subfamily profile HMM consensus sequences. Multiple sequence alignment of consensus sequences generated from profile HMMs for each of the seven MST subfamilies. Sequences were aligned using the ClustalW option in the AlignX component of the VectorNTI package. Amino acid residues highlighted in yellow indicate 100% identity across sequences. Click here for file

Additional File 11 Summary of identified MST ESTs in *Arabidopsis thaliana *EST database. Click here for file

Additional File 12 Summary of identified MST ESTs in small databases (*Marchantia polymorpha, Selaginella lepidophylla, Ceratopteris richardii*). Click here for file

Additional File 13 Summary of identified MST ESTs in *Physcometrella patens *and *Physcomitrella patens *subsp. *patens *EST databases. Click here for file

Additional File 14 Summary of identified MST ESTs in *Pinus taeda *EST database. Click here for file

Additional File 15 Summary of identified MST ESTs in *Zea mays *EST database Click here for file

Additional File 16 Summary of identified MST ESTs in *Lycopersicon esculentum *EST database Click here for file
