# Supplementary material for: The monosaccharide transporter gene family in land plants is ancient and shows differential subfamily expression and expansion across lineages
Source: BMC Evol Biol. 2006 Aug 21;6:64. doi: 10.1186/1471-2148-6-64 (PMC1578591; doi:10.1186/1471-2148-6-64)
Supplement: Additional File 1 — Chromosome map of all 53 AtMST gene loci. All 53 MST loci were mapped on the five Arabidopsis chromosomes using the Chromosome Map Tool on the TAIR website. Arrows show segmental genome duplications. Red *'s indicate two genes likely involved in a segmental duplication not detected in the TIGR genome annotation database. [file 1471-2148-6-64-S1.pdf]

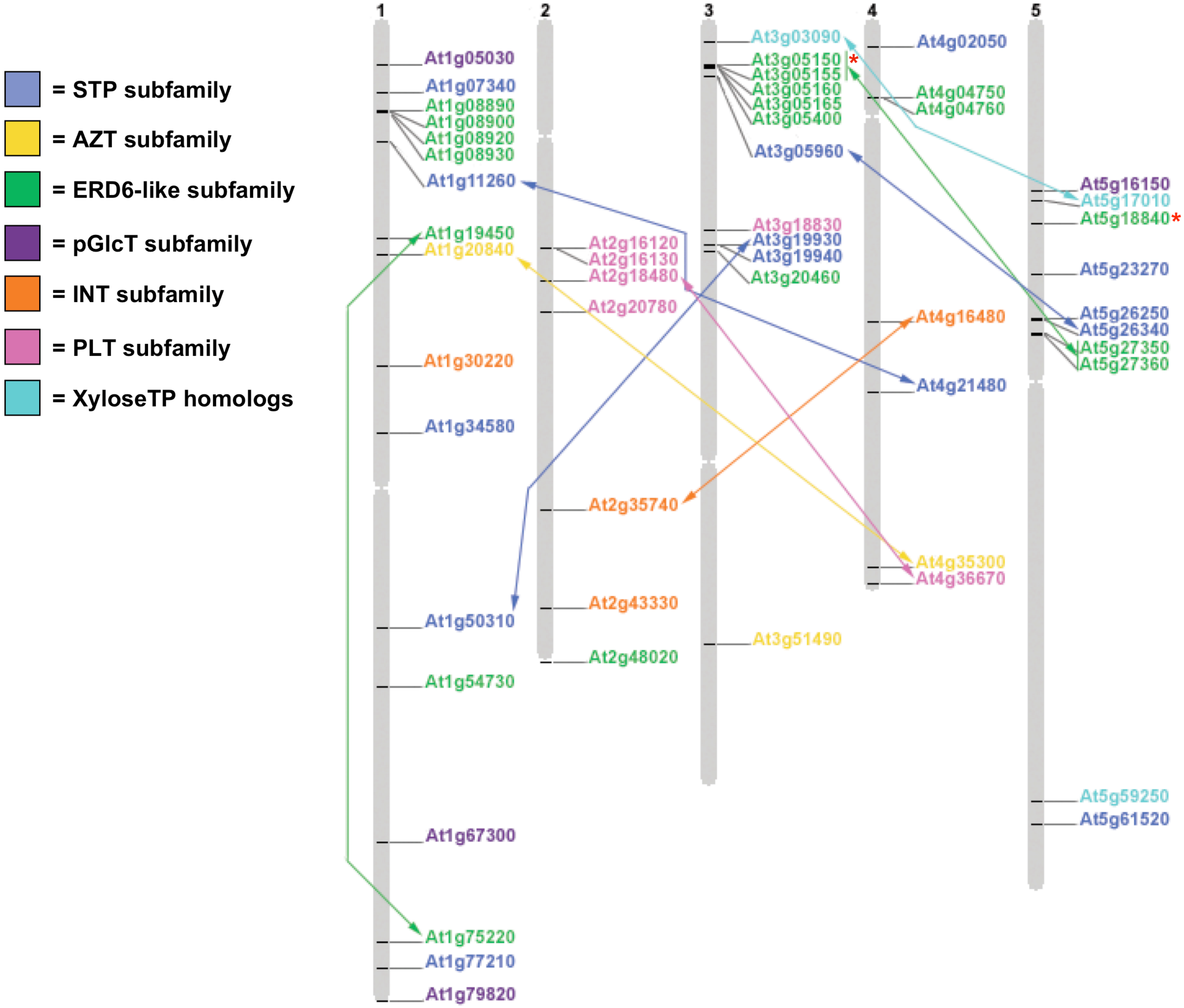
= STP subfamily
= AZT subfamily
= ERD6-like subfamily
= pGlcT subfamily
= INT subfamily
= PLT subfamily
= XyloseTP homologs
1
2
3
4
5
At1g05030
At1g07340
At1g08890
At1g08900
At1g08920
At1g08930
At1g11260
At1g19450
At1g20840
At1g30220
At1g34580
At1g50310
At1g54730
At1g67300
At1g75220
At1g77210
At1g79820
At2g16120
At2g16130
At2g18480
At2g20780
At2g35740
At2g43330
At2g48020
At3g03090
At3g05150
At3g05155
At3g05160
At3g05165
At3g05400
At3g05960
At3g18830
At3g19930
At3g19940
At3g20460
At3g51490
At4g02050
At4g04750
At4g04760
At4g16480
At4g21480
At4g35300
At4g36670
At5g16150
At5g17010
At5g18840*
At5g23270
At5g26250
At5g26340
At5g27350
At5g27360
At5g59250
At5g61520
