# Supplementary material for: The monosaccharide transporter gene family in land plants is ancient and shows differential subfamily expression and expansion across lineages
Source: BMC Evol Biol. 2006 Aug 21;6:64. doi: 10.1186/1471-2148-6-64 (PMC1578591; doi:10.1186/1471-2148-6-64)
Supplement: Additional File 2 — Pfam MST genes in viridiplantae clade used for subfamily profile HMM construction. Microsoft Word file listing all full- or nearly full-length monosaccharide transporter genes collected from the pfam database for all taxa except Arabidopsis, with SwissProt ID number, taxon and gene description listed. [file 1471-2148-6-64-S2.DOC]

| **SwissProt ID** | **Taxon** | **Description** |
| --- | --- | --- |
| Q9LLE2 | Spinacia oleracea (spinach) | hexose transporter |
| Q39416 | Beta vulgaris (sugar beet) | integral membrane protein |
| Q9LKH1 | Mesembryanthemum crystallinum | putative Na+/myo-inositol symp |
| Q9FXY8 | Mesembryanthemum crystallinum | putative glucose Translocator |
| Q9LKH2 | Mesembryanthemum crystallinum | putative Na+/myo-inositol symp |
| Q84UY4 | Mesembryanthemum crystallinum | putative Na+/myo-inositol symp |
| Q8H6S2 | Pincirus trifoliate | monosaccharide transport protein |
| Q8GTR0 | Citrus unshiu (sasuma orange) | sugar transporter |
| Q9ZR63 | Vitus vinifera (grape) | hexose transporter |
| Q9ZS76 | Vitus vinifera (grape) | hexose transporter |
| Q84N06 | Datisca glomerata (Durango root) | monosaccharide-H+ symporters |
| O04078 | Vicia faba (broad bean) | monosaccharide transport protein |
| Q7XA51 | Glycine max (soybean) | monosaccharide transporter |
| Q7XA52 | Glycine max (soybean) | monosaccharide transporter |
| Q7XA50 | Glycine max (soybean) | sorbitol-like transporter |
| Q40373 | Medicago trunculata (barrel medic) | H+/hexose cotransporter |
| Q84N02 | Medicago trunculata (barrel medic) | putative monosaccharide-H+ symporters |
| Q84QH3 | Prunus cerasus | putative sorbitol transporter |
| Q84KI7 | Prunus cerasus | sorbitol transporter |
| O48537 | Prunus armeniaca (apricot) | putative sugar transporter |
| Q84RI2 | Malus domestica (apple) | sorbitol transporter |
| Q84RI1 | Malus domestica (apple) | sorbitol transporter |
| Q9FQX3 | Apium graveolens var. dulce | mannitol transporter |
| Q8RVQ2 | Apium graveolens var. dulce | mannitol transporter |
| Q7XB36 | Orobanche ramose | putative mannitol transporter |
| Q93WT7 | Olea europaea (common olive) | hexose transporter pglt |
| Q06312 | Nicotiana tabacum | H+/monosaccharide cotransporter |
| Q9LLE1 | Nicotiana tabacum | hexose transporter |
| Q9STA8 | Lycopersicon esculentum (tomato) | hexose transporter |
| O82000 | Lycopersicon esculentum (tomato) | hexose transporter protein |
| O65322 | Petunia hybrida (petunia) | putative monosaccharide transporter 1 |
| Q8GT51 | Hordeum vulgare (barley) | sugar transporter |
| Q8GT52 | Hordeum vulgare (barley) | hexose transporter |
| Q7X9Y6 | Saccharum hybrid cultivar | putative sugar transporter type 2a |
| Q41409 | Saccharum hybrid (sugarcane) | H+/hexose cotransporter |
| Q8H6J2 | Zea mays (maize) | putative sugar transporter protein |
| Q9LLD9 | Zea mays (maize) | hexose transporter |
| Q8LJX7 | Sorghum bicolor (sorghum) | putative sugar transporter |
| Q945E5 | Oryza sativa (rice) | putative sugar transporter |
| Q9FRK0 | Oryza sativa (rice) | putative sugar transporter |
| Q9AUM9 | Oryza sativa (rice) | putative sugar transporter |
| Q852B0 | Oryza sativa (japonica cultivar) | putative sugar transporter protein |
| Q7XIZ0 | Oryza sativa (japonica cultivar) | putative proton myo-inositol transporter |
| Q7XC58 | Oryza sativa (japonica cultivar) | putative monosaccharide transporter |
| Q7XFD6 | Oryza sativa (japonica cultivar) | putative mannitol transporter protein |
| Q9FRT5 | Oryza sativa (japonica cultivar) | monosaccharide transporter 3 |
| Q851G4 | Oryza sativa (japonica cultivar) | monosaccharide transporter 2 |
| Q9AUT4 | Oryza sativa (rice) | putative hexose carrier protein |
| Q943S2 | Oryza sativa (rice) | putative hexose transporter |
| Q8LHC3 | Oryza sativa (japonica cultivar) | similar to myo-inositol transporter 2 |
| Q8H887 | Oryza sativa (japonica cultivar) | putative sugar transporter protein |
| Q7XCM9 | Oryza sativa (japonica cultivar) | putative sugar transporter |
| Q9SNK7 | Oryza sativa (rice) | similar to sugar transporter protein |
| Q9FRT6 | Oryza sativa (rice) | monosaccharide transporter 2 |
| Q9FRP7 | Oryza sativa (rice) | putative sugar transporter protein |
| Q94EC3 | Oryza sativa (rice) | putative monosaccharide transporter 3 |
| Q8W2W8 | Oryza sativa (rice) | putative mannitol transporter protein |
| Q8H4I7 | Oryza sativa (japonica cultivar) | putative hexose carrier protein hex6 |
| Q94EC4 | Oryza sativa (japonica cultivar) | putative monosaccharide transporter3 |
| Q9FRT7 | Oryza sativa (rice) | monosaccharide transporter 1 |
| Q7XBT2 | Oryza sativa (japonica cultivar) | putative sugar transporter protein |
| O24245 | Picea abies (Norway spruce) | H+/monosaccharide cotransporter |
