# Supplementary material for: The monosaccharide transporter gene family in land plants is ancient and shows differential subfamily expression and expansion across lineages
Source: BMC Evol Biol. 2006 Aug 21;6:64. doi: 10.1186/1471-2148-6-64 (PMC1578591; doi:10.1186/1471-2148-6-64)
Supplement: Additional File 10 — Alignment of MST subfamily profile HMM consensus sequences. Multiple sequence alignment of consensus sequences generated from profile HMMs for each of the seven MST subfamilies. Sequences were aligned using the ClustalW option in the AlignX component of the VectorNTI package. Amino acid residues highlighted in yellow indicate 100% identity across sequences. [file 1471-2148-6-64-S10.pdf]

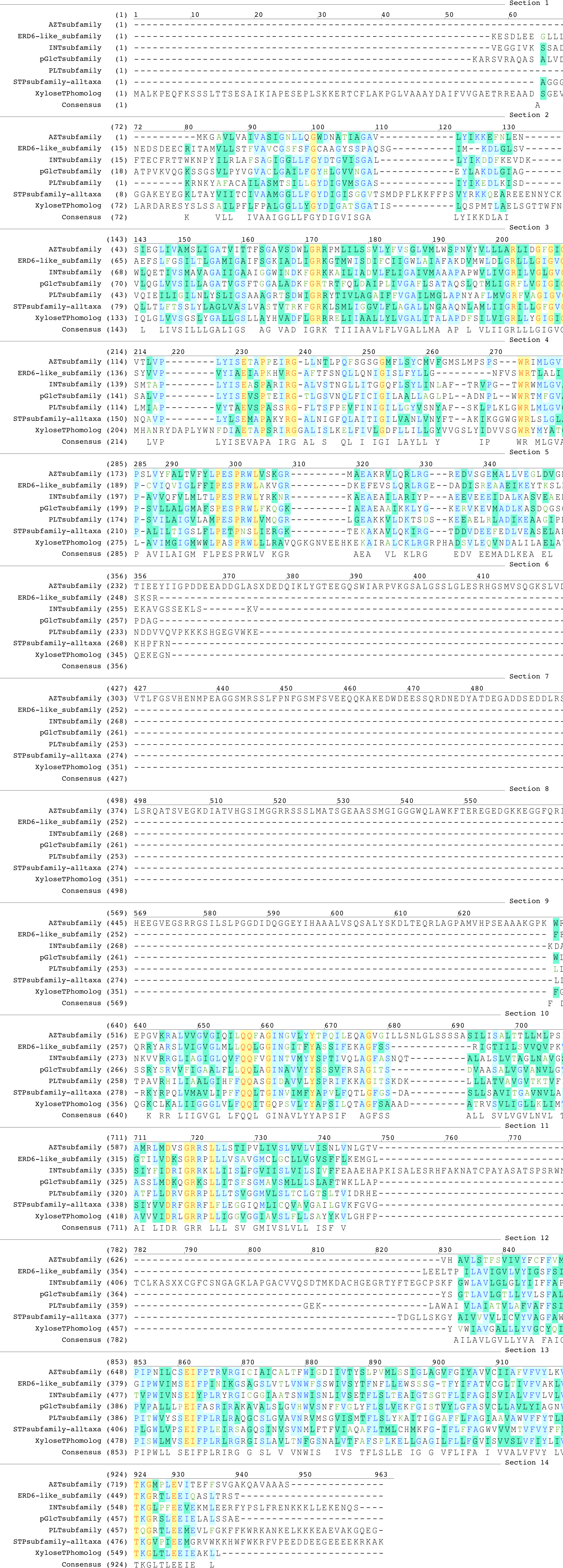

Section 1
AZTsubfamily
ERD6-like_subfamily
INTsubfamily
pGlcTsubfamily
PLTsubfamily
STPsubfamily-alltaxa
XyloseTPhomolog
Consensus
Section 2
Section 3
Section 4
Section 5
Section 6
Section 7
Section 8
Section 9
Section 10
Section 11
Section 12
Section 13
Section 14
